# Rotavirus Interactions With Host Intestinal Epithelial Cells

**DOI:** 10.3389/fimmu.2021.793841

**Published:** 2021-12-22

**Authors:** Joshua Oluoch Amimo, Sergei Alekseevich Raev, Juliet Chepngeno, Alfred Omwando Mainga, Yusheng Guo, Linda Saif, Anastasia N. Vlasova

**Affiliations:** ^1^ Center for Food Animal Health, Department of Animal Sciences, College of Food Agricultural and Environmental Sciences, The Ohio State University, Wooster, OH, United States; ^2^ Department of Animal Production, Faculty of Veterinary Medicine, University of Nairobi, Nairobi, Kenya; ^3^ Department of Public Health, Pharmacology and Toxicology, Faculty of Veterinary Medicine, University of Nairobi, Nairobi, Kenya

**Keywords:** immunity, intestinal epithelial cells, immune receptors, rotaviruses pathogenesis, immune evasion

## Abstract

Rotavirus (RV) is the foremost enteric pathogen associated with severe diarrheal illness in young children (<5years) and animals worldwide. RV primarily infects mature enterocytes in the intestinal epithelium causing villus atrophy, enhanced epithelial cell turnover and apoptosis. Intestinal epithelial cells (IECs) being the first physical barrier against RV infection employs a range of innate immune strategies to counteract RVs invasion, including mucus production, toll-like receptor signaling and cytokine/chemokine production. Conversely, RVs have evolved numerous mechanisms to escape/subvert host immunity, seizing translation machinery of the host for effective replication and transmission. RV cell entry process involve penetration through the outer mucus layer, interaction with cell surface molecules and intestinal microbiota before reaching the IECs. For successful cell attachment and entry, RVs use sialic acid, histo-blood group antigens, heat shock cognate protein 70 and cell-surface integrins as attachment factors and/or (co)-receptors. In this review, a comprehensive summary of the existing knowledge of mechanisms underlying RV-IECs interactions, including the role of gut microbiota, during RV infection is presented. Understanding these mechanisms is imperative for developing efficacious strategies to control RV infections, including development of antiviral therapies and vaccines that target specific immune system antagonists within IECs.

## Introduction

Rotaviruses are the topmost pathogens implicated in acute diarrhea in children and in young animals globally. Rotaviruses belong to *Reoviridae* family and are nonenveloped viruses having three layers of proteins that contains 11 segments of genomic dsRNA, encoding six structural (VP1–6) and six nonstructural (NSP1–6) proteins (1). RV infects small intestinal mature enterocytes and enteroendocrine cells leading to disruption of the intestinal epithelial cell (IEC) homeostasis due to villus atrophy, increased epithelial cell turnover, enhanced apoptosis, and formation of large vacuoles in enterocytes (2–5). IECs serve as the first physical barrier against RV infection. In addition to being a physical barrier, IECs produces mucus and cytokine/chemokines, including toll like receptor (TLR) expression and signaling to reduce risk of RV infection (6–8). We discuss these mechanisms in detail in various sections of this review. Since RVs rely on host cells for their replication and transmission, they have evolved numerous strategies to escape and/or impair the host immunity (9). The attachment of RVs to the membrane of host enterocytes requires the presence of specific glycans such as mucin, histo-blood group antigens (HBGAs) and sialic acids (SA) (10–12). Some of the mechanisms employed by RVs to evade host immune responses include (i) degradation of IFN-regulatory factors (13–15); (ii) inhibition of nuclear accumulation of STAT1, STAT2 (16) and nuclear factor κB (17, 18); (iii) formation of vesicle-cloaked virus clusters (19, 20); and (iv) induction of intracellular calcium waves through adenosine diphosphate (ADP) signaling (21). We present a summary of the current knowledge on the RV-IECs interactions that influence host immunity and pathogenesis of RV infection.

## Rotavirus Cell Entry

**Figure 1:**
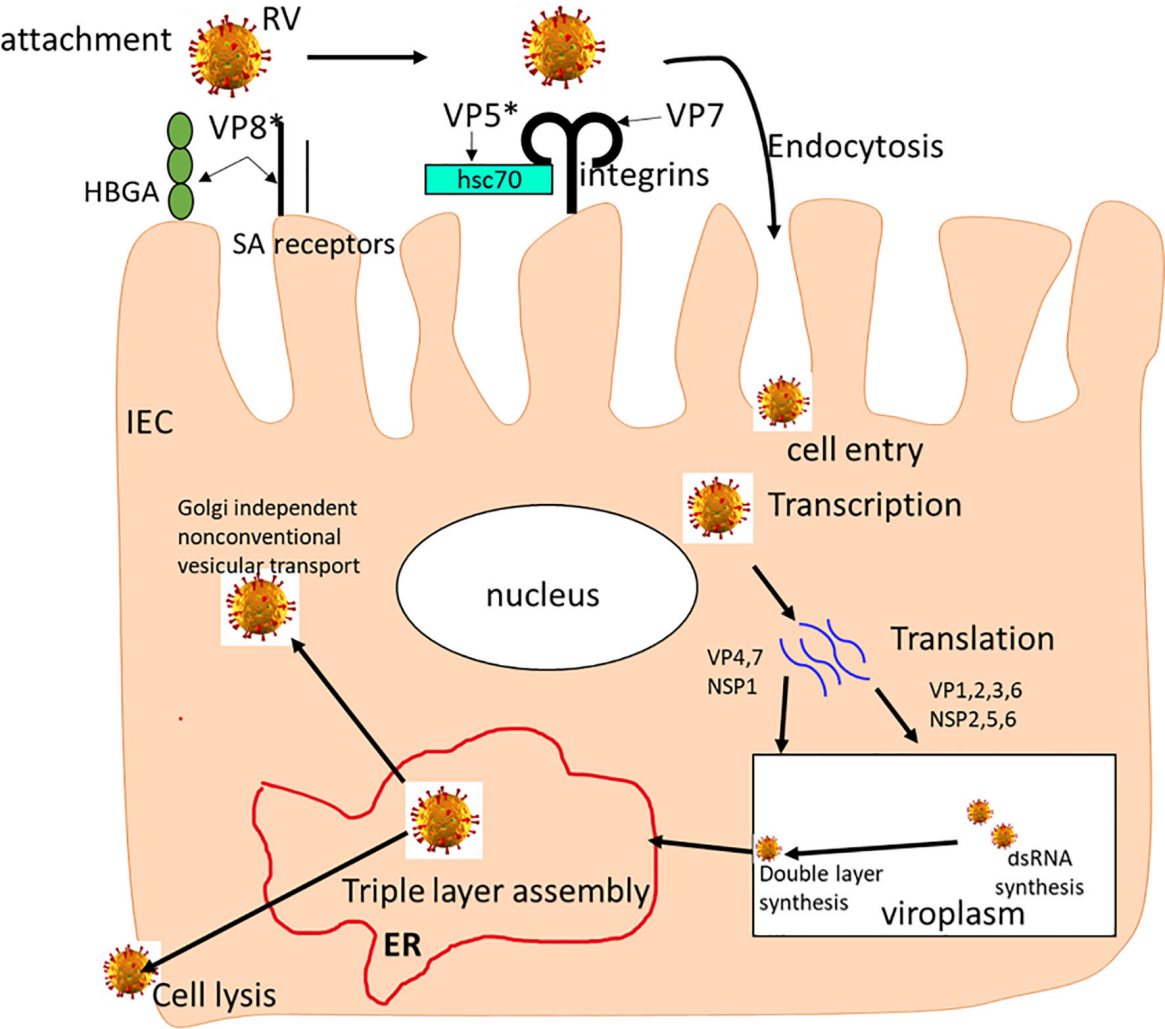
The rotavirus replication cycle. The RV attaches to SAs and HBGAs on the host cell surface, then interacts with integrins and Hsc70 receptors where it is internalized by receptor-mediated endocytosis. Removal of the outer layer results in the release of double-layered particles (DLPs) in the cytoplasm where it undergoes mRNA transcription. mRNAs are translated into viral proteins, replicated and packaged viroplasms as DLPs. Then, the DLPs bind to NSP4 and bud into the endoplasmic reticulum (ER) where transient membranes are removed as VP4 and VP7 proteins assemble, leading to maturation of the TLP virion. The newly formed TLP virions are removed through cell lysis and in polarized epithelial cells, through Golgi-independent non-classical vesicular transport mechanism.

Upon entry in the body, viruses infect mucosal epithelial cells and replicate in cell cytosol. There are two pathways of viral cell entry including direct entry at the epithelial plasma membrane, or *via* epithelial endocytosis (22, 23). Thus, the molecular interactions between the virus and target-cell receptors influences the viral entry pathway. RVs cell entry process consists of proteolytic priming, cell attachment, digestion of the outer capsid, and internalization of the RV double-layer particles (DLPs) into the cytoplasm (**Figure 1**). RVs require interactions between VP7 and cleaved VP4 to initiate cell entry process (23–28). The VP4 protein is cleaved into VP8* and VP5* domains, with each domain playing a distinct role in the cell entry process (29–32). The direct membrane-penetration pathway was revealed by the study of Denisova and colleagues where they observed that trypsinized RV induces rapid release of Cr^51^ from cells facilitated by the VP5* cleavage product which permeabilizes lipid vesicles *in vitro*. Therefore, VP5* is a specific membrane-permeabilizing capsid protein involved in RV cell entry (33). On the other hand, receptor-mediated endocytosis relies on the acidification of endosomes leading to partial uncoating or entry into the cell (10); however, RV cell entry by direct penetration is not inhibited by lysosomotropic agents or endocytosis inhibitors (34). Irrespective of the entry pathway, the RV entry mechanism has been reported to involve both membrane permeabilization (33–35) and uncoating of the outer capsid layer (36, 37).

As shown in **Figure 1**, many cell surface molecules, including SA, HBGAs, hsc70 and integrins, are believed to play a role during early RV-IEC interactions, implying that SA-dependent or SA-independent RVs use common steps during binding and/or entry into the host cells (27, 38–40). These initial studies and many subsequent studies revealed that SA-dependent and SA-independent RV cell entry mechanisms differ and most likely uses different receptors. There are many host factors that affect RV entry into the cell, replication and virus release. As illustrated in **Figure 1**, during RV release, RV particles can be assembled in five steps., (i) RV particles relocate from the endoplasmic reticulum (ER) to the apical plasma membrane bypassing the Golgi complex; (ii) RV NSP4 protein halts the early secretory pathway and arrests normal ER-to-Golgi membrane trafficking; (iii) VP4 and VP7 are detected in a filamentous array; (iv) VP4 present at the plasma membrane associated with microtubules; and (v) interaction of NSP4 and lipid membranes mimicking endocytosis. Most recently, Herrmann and colleagues using electron cryomicroscopy demonstrated the involvement of VP4 gene in the initial RV penetration into the membranes of target cells (41). They showed that when VP4 is cleaved into VP5* and VP8*, it transitions from an upright to a reversed conformation leading to membrane perforation and virion attachment. Together, this shows that interactions between RV and cellular proteins regulates virus morphogenesis and release, *via* activating cell signaling pathways.

## Rotavirus Interactions With Intestinal Epithelial Cells

RV mainly targets the terminally differentiated IECs, primarily of the ileum and jejunum (42), and thus IECs serve as the first physical barrier. Besides physical barrier, IECs employ several innate immune mechanisms to inhibit infections with enteric pathogens. The mechanisms epithelial cells employ to counteract RV infection involve mucus production, secretion of cytokine/chemokine, and TLR expression and signaling (8, 43). The first step in RV infection is the attachment and entry into the target cells using cell surface molecules, such as SA, HBGAs, Hsc70 and integrins, as receptors or co-receptors (**Figure 1**) (44–50). Rotavirus attachment to enterocytes occurs in the presence of specific glycans including mucin glycans and cell surface glycans (HBGAs and SA) (10–12). While the host glycan specificity is one of the key factors regulating RV infectivity, other factors are also thought to influence relative prevalence and emergence of different RV genotypes across various populations. Such factors may include but are not limited to variable glycan recognition and binding affinities by RVs of different genotypes, the existence or lack of additional co-receptors/co-factors, disparity in immune responses, and other undefined host factors (51). Previous work has demonstrated that the variations in glycan recognition mechanisms coincides with differences in RV species tropism, zoonotic potential, adaptation and epidemiological prevalence. Recently, we demonstrated that variations in host HBGA phenotypes are unlikely to affect the efficacy of live attenuated RV vaccines, since attenuation by cell culture adaptation decreases genotype-specific HBGA selectivity of some RV strains (44).

### Rotavirus Interactions With Mucins

Mucins are high molecular weight products of epithelial cells of high molecular weight. These heavily glycosylated proteins are key components of mucus. Mucins function as chemical barriers as well as binding pathogens as part of the innate immune system. The O-glycan structures in mucin are diverse with 8 recognized cores where core 1-4 is commonly found in intestinal mucins (52). Core 1 and core 2 structures are commonly found in gastric and duodenal mucins, core 3 structure predominate in the small intestinal mucins, while core 3 and 4 structures form the majority of colonic mucins (53–55). Rotavirus has been reported to bind to core 2 and core 4 mucin structures, located in the intestine (56). The binding was observed to be conserved across multiple RV strains (57, 58). For example, human RV (HRV) P[8] and P[4] is reported to recognize mucin cores 2, and 6 (59). Sun et al. (60) observed that porcine but not human P[6] binds to mucin core 2, while both human and porcine P[19] and rare P[10] can bind to mucin core 2 (58, 60). Most recently, Engevik and colleagues (2) observed that RV binding to MA104 cells is inhibited by supplementation of murine intestinal mucin, which is suggestive of molecular mimicry of mucins allowing them to interfere with virus-cell attachment. However, they showed that neuraminidase treatment which removes murine SAs inhibited the ability of mucins to prevent RV attachment to host cells, indicating the functional significance of mucin polymeric structure and SA content in its induction role. Earlier, Boshuizen and colleagues demonstrated that mucus from mock control mice neutralized infection by the HRV (prototype Wa strain) in Caco-2 cells, while mucus from murine RV infected mice was less effective in inhibiting RV infection (61). In the ileum, abundance of glycan-degrading bacteria is favored by the availability of glycans produced in response to RV infection, which leads to further reduction in mucin-protection against RV infection. For example, mucin-degrading bacteria has the ability to come closer to the host epithelium than non-mucin-degrading bacteria, thus indirectly facilitating RV attachment to epithelium. Taken together, these findings suggest that RV may stimulate mucin production, especially core 2, to enhance infection and interspecies transmission (2). In summary, the mucin structure seems to be an important host barrier to RVs. Therefore, in-depth understanding of RV interaction with mucin glycans may provide additional insights into RV adaptation, transmission, and host restriction which may help in developing targeted therapeutics for RV infection.

### Rotavirus Interaction With Sialic Acids

Sialic acids (SA) were the first recognized RV receptors involved in the early cell attachment *via* their interactions with VP4 spike protein (VP8* domain) (46). Sialic acids are present in swine small and large intestinal epithelium, with colonic crypts showing a greater abundance than the small intestine (62). Studies have shown that neuraminidase (sialidase) treatment affects some RVs (mainly animal RVs) infectivity in cell culture, and that sialylated glycoproteins of variable origin inhibits infection with these strains (50, 63–65). Similar to other viruses including influenza A virus, reovirus type 3, various coronaviruses and Sendai virus, the RV ability to agglutinate red blood cell (RBC) was SA-mediated. Some RVs, mostly human strains, did not cause RBCs agglutination and hence neuraminidase treatment did not alter or increased RV infectivity in cell culture. This led to the categorization of RVs as neuraminidase (Neu)-sensitive and Neu-insensitive with the entry pathway SA-dependent and SA-independent, respectively (38, 66). RVs are classified based on the genetic/antigenic variability in their VP4 gene [Protease sensitive (P genotype)]. Currently there are 57 genotypes (P[1-57] of RVs of group A. Human and animal RVs (ARVs) belonging to P genotypes [1], [2], [3], and [7] are SA-dependent while all HRV and ARV strains of the remaining known P genotypes are SA-independent (67). However, this classification was challenged by some authors who argued that the lack of sensitivity to neuraminidase treatment does not imply that the entry pathway is SA-independent (68, 69). These authors supported their argument by demonstrating that even though the Neu-sensitive RV strains recognize the terminal SA moieties, the Neu-insensitive RV strains also attach to internal SA moieties in gangliosides that are resistant to neuraminidase treatment. Therefore, these authors recommended a more vigorous classification based on ganglioside specificity rather than neuraminidase sensitivity (69, 70).

The absence of a defined receptor for the Neu-insensitive RVs triggered further research that led to discoveries of other potential attachment factors and (co) receptors, including: (a) the Hsc70 (26, 71); (b) gangliosides (68, 72, 73); (c) integrins (25, 74, 75); and (d) HBGAs (45, 76–78). Haselhorst and colleagues (69), using nuclear magnetic resonance (NMR) spectroscopy reported a direct interaction between a Neu-insensitive human RV prototype Wa and SAs, which was contrary to the widely accepted paradigm that SAs are not relevant in host cell recognition by the Neu-insensitive RVs.

Regardless of neuraminidase sensitivity, studies have demonstrated that both Neu-sensitive and Neu-insensitive RVs uses VP8* domain of VP4 during the attachment to SA moiety in the gangliosides (69, 79, 80). Ciarlet and co-workers showed differences in cell entry between the two classes of RVs, where they demonstrated that, in differentiating epithelial cells, Neu-sensitive RVs enter only through the apical surface, while the Neu-insensitive RVs enter through both apical and basolateral surfaces (67). Additionally, significant differences in distribution of escape mutations selected with VP8*- and VP5*-specific neutralizing antibodies in the two classes of RV has been observed (66, 81). For example, for the Neu-sensitive RVs, such antibodies largely map to the VP8* domain whereas for Neu-insensitive RVs they map mostly to VP5* domain with less mutations in VP8* domain. Moreover, Ciarlet and colleagues further demonstrated that the there was no change in reassortant RVs (having alteration in VP7-VP4 combinations) for SA residue requirement for effective infection (67). Hence, in addition to being a prerequisite for infectivity, the existence of SA on the epithelial cell surface is necessary for efficient RV growth in cell culture. Most recently, we demonstrated that differences in HBGA-RV and SA-RV interactions determine replication efficiency of virulent group A RVs (P[8], P[5], P[6] and P[13] genotypes) in porcine small intestinal enteroids (PIEs) (44). In this study we demonstrated that inhibition of HBGAs synthesis decreases HRV Wa replication, while it only slightly affected ARVs (OSU and G9P[13]) replication, indicating that the selected HRVs and ARVs employ different attachment factors for infection (44). Additionally, we confirmed the previous findings that neuraminidase treatment strongly inhibits the growth of OSU (Neu-sensitive) and demonstrated for the first time that G9P[13] replication (Neu-dependent) was significantly enhanced by neuraminidase treatment, hence, other receptors recognized by G9P[13] may become unmasked after removal of terminal SA (69). Recently, Ding et al. (82) using CRISPR/Cas9 screening strategy identified SLC35A1 gene which is critical in RV infection. SLC35A1 gene is a key Golgi-resident glycosyltransferase essential for sialic acid synthesis. These findings led to our conclusion that genetically distinct RVs have developed various strategies for successful cell attachment and entry. Overall, these studies demonstrated the following: (i) that RVs cell entry process require VP5*; (ii) that multiple cell attachment factors exist; and (iii) that differences exist in cell entry pathways between Neu-sensitive and Neu-insensitive RV strains. This indicates that RV cell entry pathway is complex and involves many receptors (26). The Neu-insensitive HRVs recognizes HBGAs as a different attachment factor or receptors, possibly having similar function as the terminal sialic acid for Neu-sensitive ARVs. Thus, whether SA act coordinately with HBGAs or they represent independent different routes for RV entry remains to be determined.

### Rotavirus Interactions With Integrins

Integrins, heterodimeric transmembrane receptors, are utilized by several viruses for successful attachment and infection of epithelial cells. Several integrins, (such as α2β1, α4β1, αxβ2, and αvβ3), have been described as potential receptors for RV cell entry (25, 39, 40, 74, 83). In the cell culture and gut tissues, some integrins are expressed basolaterally while others such as α2β1, are expressed apically in crypt and villus enterocytes. Moreover, studies have demonstrated that RV infectivity is moderately inhibited by peptides containing the ligand motifs and monoclonal antibodies to these integrins, an indication that these integrins may play a role as RV (co)receptors or attachment factors (24, 40, 75, 84). Therefore, interaction between RV and integrins may be needed for initiating events necessary for viral cell entry and establishing infection (85). Upon expressing these integrins *via* transfection of poorly tolerant cells, Hewish and colleagues observed increased attachment and infection, further supporting the involvement of integrins in RV cell entry (75). Finally, multiple studies have shown that both VP5* domain of VP4 and VP7 are involved in interactions with integrins during RV cell entry (25, 74, 83, 86, 87). Altogether, RV binding to integrin facilitates virus-cell membrane interactions which induce the signal for virus internalization, and therefore a full understanding of the interactions between RV and integrins and their importance for viral pathogenesis is critical for novel therapeutics based on cell entry inhibition.

### Rotavirus Interaction With Heat Shock Cognate Protein

Heat shock proteins (HSPs) are a family of highly homologous molecules protecting cells from damage caused by various physical and chemical stresses, including pathogen invasion. The heat shock cognate protein (hsc70) is 70 kDa in size and is highly conserved in all organisms (88). Numerous *in vitro* studies have demonstrated expression of hsc70 on the cytoplasmic membrane of many cell lines, including MA104, Caco-2, BHK (89), B cells (90), IECs (91), and that hsc70 is involved in virus invasion as a receptor or co-receptor [reviewed in (92)]. On the other hand, hsc70 also plays a role as an antagonist to destroy virus by enhancing cellular antiviral innate and adaptive immunity. For example, besides SA-containing receptors and integrins, hsc70 interacts with RV after early attachment phase of cell entry, a process facilitated by VP5* domain of VP4 spike protein (71, 89). Additionally, Gualtero and colleagues (93) reported an interaction between RV DLPs with hsc70 and revealed indirect role of VP6 in RV cell entry. In summary, studies have shown that RVs interacts in multiple ways with hsc70, involving VP4 and VP6, and together with integrins and other cell surface glycans extends the range of potential optional cell-binding and entry mechanisms, facilitating RV infection of diverse hosts and cell types. Therefore, an in-depth understanding of the mechanisms of hsc70-RV interactions is essential to define the hsc70 role in RV pathogenesis and the feasibility of its use as a drug development target.

### Rotavirus Interaction With Histo-Blood Group Antigens

Histo-blood group antigens are polymorphic complex carbohydrates found on RBCs and mucosal epithelia surfaces of the reproductive/urinary, respiratory and gastrointestinal tracts (94, 95). HBGAs are also present in other biological fluids like intestinal contents, saliva and milk of secretor positive individuals (96). They are formed through addition of monosaccharides to disaccharides, a process catalyzed by glycosyltransferases encoded by ABO (H), secretor (FUT2) and Lewis (FUT3) (96). One important role of the HBGAs synthesized by the ABO, Lewis and secretor genes would be to provide receptors for pathogens. However, since HBGA-like structures may also be present on the pathogens, antibodies against HBGAs may be generated by the host, hence ABO, Lewis and secretor genes also function to provide polymorphism within the species to cope with diverse and rapidly evolving pathogens. Haga and coauthors using human intestinal enteroids demonstrated that secretor status, defined by FUT2 gene expression, is essential to support HuNoV replication (97). They showed that for successful HuNoV infection, fucosylation of HBGAs is crucial for initial binding and modification of other receptors needed for viral uptake and uncoating. Therefore, to determine the role of FUT2 in rotavirus replication, genetic deletion of FUT2 in human/porcine small intestinal enteroids will be an invaluable tool.

HBGAs possess different ABO, Lewis and secretor versus non-secretor types ubiquitous among world populations. Comparable diverse HBGAs have been reported in most animals, based on their ABO, secretor and Lewis families status, leading to conclusion that human and animals share HBGA products. HBGAs are richly distributed on intestinal epithelial mucosa, where they act as attachment factors or receptors for many enteric pathogens, including human norovirus (HuNoVs), RVs, and *H. pylori* (98, 99). Since RVs are genotypically diverse, they recognize HBGAs in a genotype-specific manner (100, 101), contributing to genotype-specific RV host ranges among different populations. Therefore, RV cross-species transmission between humans and animals could be due to these shared HBGAs. Jiang and co-authors presented a comprehensive review of HBGAs as receptors for RV, and how that impacts RV epidemiology, disease burden and vaccination strategies (47).

The host specificity, tissue tropism, and zoonotic potential of RVs are determined by the nature of RV attachment to cell surface glycan receptors (102). Studies have demonstrated that majority of ARVs and nearly all HRVs are Neu-insensitive and therefore SA-independent, although HRV prototype (Wa) is reported to recognize an internal SA moiety (38, 67, 69). Additionally, it has been shown that nearly all P genotypes in genogroups P[II]–P[IV], that frequently infect humans, recognize HBGAs leading to the hypothesis that HBGAs are essential attachment factors or (co) receptors for RVs (45, 76–78).

HBGAs type 1 recognize RVs P[4,6 and 8] human genotypes which cause >90% of RV infections in children globally. Recent studies have elucidated the importance of HBGAs as cell attachment and susceptibility factors for these worldwide dominant human RV (44, 60, 103). HBGAs have been shown to be potential factors for RV cross-species transmission. For example, oligosaccharide-binding experiments revealed attachment of RVs genogroup P[III] (comprising P[9], P[14], P[3] and P[25]) to A antigens (45, 76), whereas their recombinant VP8*s also attached human and animal mucins with a positive correlation between the binding signals and A antigens (76). These findings imply that the shared A antigen between humans and animal species may play a role in the observed cross-species transmission of P[III] genogroup RVs. The VP8* of P[II] genogroup RVs (including the worldwide dominant P[4], P[6], and P[8] strains and the rare P[19] genotype), recognizes H-type I HBGA together with mucin cores (59, 98). Recently, Hu and co-authors reported a unique glycan binding site in the crystal structures of P[4] and a neonate-specific P[6] VP8*s alone and in complex with H-type I HBGA, allowing for the binding of ABH HBGAs, consistent with their worldwide dominance (103). Additionally, same authors showed that the VP8* of neonatal P[6] RVs exhibits slight structural changes in this binding site restricting its ability to bind branched glycans. Furthermore, Nordgren and colleagues showed that VP8* P[6] preferentially infect Lewis-negative children (77).

RVs utilizes the VP8* to recognize specific host glycans in a genotype-dependent manner (101, 104), as has been shown in recent studies. For example, Liu et al. demonstrated that VP8* of P[19] attached to type I glycans at a site distinct from the previously described A-type or precursor binding sites in RVs of P[14] and P[11] genotypes (57, 59). Bohm and colleagues showed in their study that P[4] and P[6] HRVs an bind to A-type HBGA, while P[8] does not (105). In sharp contrast to these findings, Perez-Ortin and colleagues demonstrated that children with blood groups A and AB were much more susceptible to RV P[8] gastroenteritis (106). Additionally, several epidemiological studies in different countries and continents have shown that P[8] RV genotypes predominantly infected secretor types (99%) (48, 78, 106–109). Recent studies have also demonstrated that Le^b^ and secretor status are important host susceptibility factors for the infection of P[8]/P[4] RVs (106, 110, 111). Moreover, we recently demonstrated that inhibition of HBGAs synthesis reduces HRV Wa (G1P[8]) replication, further suggesting that replication of HRV Wa strain may need HBGA as attachment factor or receptor (44). Resemblances in the glycan specificity and attachment between the global dominant HRVs (P[4], P[6], P[8]) and the rare P[19] RV, which also commonly infects pigs, perhaps signifies a zoonotically related evolution of these strains (103).

Even though majority of animal RVs recognize sialoglycans, some of them and nearly all human RVs exclusively bind to HBGAs on IECs and in mucosal secretions (45, 59, 69, 105, 112, 113). Recent studies have demonstrated that the severity of RV infection greatly correlates with the secretor status of the individual, indicating that the HBGAs are cell attachment and susceptibility factors for HRVs (77, 78, 108, 114). Previous crystallographic studies revealed that the VP8* of the P[14] and P[25] HRVs bind to A-type HBGA at a site overlapping with the SA binding site in ARVs, providing a new understanding of inter-species transmission of an animal-origin virus to the human (45, 115). on the other hand, a neonate-specific bovine-human reassortant P[11] HRV VP8* was shown to recognize type I and type II precursor glycans expressed in the neonatal intestine and human milk, while its bovine counterpart only binds to type II glycans (112), coherent with their large quantity in bovine milk.

RVs-and HBGAs interactions have been reported by numerous studies, including: (i) *in vitro* attachment of recombinant RV surface spike protein VP8* with the A, B, H (secretor) and Lewis antigens from human saliva, milk, synthetic oligosaccharides and RBCs (hemagglutination); (ii) glycan array analyses of RV–HBGA interactions; (iii) X-ray crystallography and STD NMR analyses to resolve the atomic structures of VP8* proteins for select human RVs (P[14], P[11] and P[19]), in complex with their HBGA oligosaccharide ligands (59, 103, 112, 116, 117). Recently, Sun et al. showed that porcine P[6] strain (z84) have a similar structure as P[19] VP8* (60). However, the same authors observed that HRV P[6] and P[19] recognizes H-type I HBGA, whereas porcine P[6] and P[19] do not. The specificities of RV interaction with HBGAs were demonstrated through the binding of triple layer RVs, but not the double layer particles, further supporting the significance of the VP8* in RV-host interaction during RV infection.

Intestinal enteroids (IEs) which naturally express HBGAs are invaluable *in vitro* model which could be used to study the HBGA-RV interactions (114). There are selective interactions between virulent RVs and HBGAs expressed by Porcine IEs. Most recently, we showed that replication efficacy of virulent group A RVs in PIEs depend on HBGAs-RV and SA-RV interactions (44). For example, porcine G5P[7] (OSU) and G9P[13] strains showed a strong preference for the H^+^ PIEs. Since, both P[7] and P[13] belong to the P [I] genogroup of RV, it implies that P[I] porcine RVs selectively recognize H-type1 HBGA, however, the receptors for RV cell entry seems to be redundant since the OSU strain has been shown to be both SA and H dependent. Further, *in vitro* experiment confirmed that most attenuated RVs lose HBGA selectivity when there is lack of HBGA expression by MA-104 cells (44),. Moreover, attenuated Wa (G1P[8]) and Gottfried (G4P[6]) RV strains showed reduced replication ability in the PIEs while attenuated G9P[13] and OSU (G5P[7]) strains efficiently replicated in PIEs, suggesting variability of binding efficiency of attenuated strains (44). However, since RVs interact with the hsc70 and integrins during initial cell entry process (10), partial homology of these proteins shared between different species could lead to variable levels of RV replication in IEs.

Unlike group A RVs (RVA), the current knowledge of other groups of RVs (B-J), in terms of host range, epidemiology, evolution, interspecies transmission, and pathogenesis, is limited. Furthermore, the majority of these RV groups grow poorly or do not grow at all in cell cultures rendering further *in vitro* studies very difficult. Previously, Svensson reported that RVC possesses a hemagglutinin which requires SAs for RBC and cell receptor binding (118). Most recently, using glycan microarrays, Sun and colleagues showed that the human group C RV (RVC) VP8* recognizes type A HBGA but not type B and H, and that the VP8*-HBGAs interaction mechanism utilized by RVC is distinctive from that used by RVAs (119). Human infections with RVB have been reported, especially in Asia (120, 121), however, their receptor binding specificity is still not well understood. Thus, more studies are imperative to elucidate mechanisms of cell entry and replication of other groups of RVs.

In summary, the new insights into HBGA-controlled RV host ranges, evolution and epidemiology highlights the significance of the VP8* in RV pathogenesis and transmission. The recognition of HBGAs as RV receptors provides further useful understandings into the performances of existing licensed RV vaccines in different populations and emphasizes development of a vaccine based on P-types “personalized vaccines”, which can provide protection against the specific genotypes that dominate in certain populations.

### Role of Gut Microbiota in RV-IECs Interactions

The tripartite interactions between host epithelial cells, RVs and the gut microbiome are important in maintenance of gut health. The dual regulatory effects of gut microbiota on viral infection has been demonstrated by several studies, i.e., they *facilitate* viral infection, and *inhibi*t viral infection through numerous mechanisms [reviewed by Yang et al. (122)]. Some of the mechanisms that have been proposed include: (i) colonization of the intestinal epithelium resulting in decreased pathogen attachment; (ii) bacterial binding to IEC receptors which reduces viral attachment and entry; and (iii) stimulatory effects on the host immune system *via* alteration of the intracellular transcription and translation (123, 124). Some researchers have hypothesized that the gut microbiota may influence RV infection thereby affecting the immunogenicity and protective efficacy of live oral vaccines (125). However, the microbiome role in regulation of RV infection is yet to be fully evaluated. Secretion of mucus or synthesis of potential antiviral compounds have been shown to be regulated by the enteric microbiota; thus, commensal microbes in the gut may influence the intestinal mucosa glycosylation patterns and status (126). Recently, lipopolysaccharides (LPS) were observed to bind with numerous enteric viruses thereby enhancing their environmental stability (127–130).

Mucus is the first barrier protecting IECs from being accessed by pathogens including viruses and bacteria. While the inner mucus layer, firmly adherent to IEC, is known to prevent bacterial penetration, the outer layer, loosely adherent to IEC, is a substrate for bacterial adhesion (131, 132). Most bacterial interactions with IEC have been demonstrated by *in vitro* experiments. Even though several cell lines used in some *in vitro* experiments, such IPEC-J2, are known to produce mucins, the *in vitro* models are not able to fully simulate the double mucus layer as occurring *in vivo* (133). Mucus layers are considered as the main area where bacteria interact with RVs. Mucus consists of mucin glycans that comprise highly glycosylated proteins such as HBGA and sialic acid-containing O-glycans, known to be attachment factors or receptors for RV binding (134). Intestinal mucus plays a role as “decoy” epitopes leading to elimination of viral particles from the gut lumen during the mucus clearance process (135). However, studies have shown that expelled RV particles during mucus clearance could be a source of infection of naïve individuals, hence, the presence of intestinal mucus may possibly have affect on RV transmission.

SA terminal monosaccharide is a proven target for bacterial adhesion. Certain members of *Lactobaccillus* and *Bacteroides* families bind to O-linked glycans *via* mucus-binding cell surface proteins associated with bacterial pili and flagella (136). Recently, *Bacteroides thetaiotaomicron* and *Akkermansia mucinphila* were found to degrade these SA within the mucus layer, diminishing the protective role of the mucins (2, 137, 138). For example, Shi and co-authors recently showed that a microbiome with high levels of *Akkermansia* aids RV infection, however, segmented filamentous bacteria can protect against RV infection (139). In contrast, several *Lactobacillus* species have not demonstrated the ability to degrade SA, probably due to the absence or bearing the smaller number of gene copies of glycosyl hydrolase (GH) enzymes (2).

RV was shown to bind to Gram-negative *E. coli* Nissle, and this adhesion has been proposed as one of the key mechanisms reducing attachment of RV to target cells (140). In addition to using SA as an energy source, some bacteria, such as strains of *E. coli* and *P. multocida* are known to possess cytidine 5′-monophosphate (CMP)-Neu5Ac synthetases enzymes responsible for SA biosynthesis (141). Although the presence of SA on the bacterial cell surface allows bacteria to remain undetected by the host immune system, the role of SA-producing bacteria in modulation of RV infection remains unclear.

Bacterial binding to HBGAs on the IEC surface is known as a virulence factor for some pathogenic bacteria, such as *H. pylori* (142). However, beneficial probiotic lactic acid bacteria such as *Lactobacillus brevis* has been found to interact with human blood type-A antigen *via* sugar specific protein lectins (101, 143). Studies have shown that *E. cloacae* produces HBGA-like substances that are capable of binding to RV *via* interaction with the spike protein VP8* (101). An *In vivo* experiment has demonstrated reduced human NoV shedding in *E. cloacae* colonized pigs (144, 145).

In addition to binding RVs, integrins have been found to interact with *H. pylori* adhesin, an outer membrane protein (OMP), that binds to several integrin heterodimers including α4β1 (146). There is an αvβ3-integrin-binding protein OMP P66 secreted by *B. burgdorferi* that provides the adherence to host cells ( (147). Additionally, the Hsc70 has been identified as a component of the host cell that is utilized by several pathogens such as *E. coli*, *S. typhimurium* and *L. monocytogenes* (148). Presence of integrin-binding factors on beneficial bacteria and/or their capability to interact with Hsc70 have not yet been identified. Recent studies have demonstrated that proteins in probiotic extracts blocks viral adhesion, but not permeation, thereby inhibiting RRV strain infection of MA104 cells, and that Hsc70 and β3-integrin are the receptors involved in this blockage mechanism (49, 149). Laino and colleagues showed in their study that some probiotic bacteria, such as *Lactobacillus delbrueckii*, produces immunomodulatory extracellular polysaccharides (EPSs) which allow the crosstalk between bacteria and host IECs through interaction with pattern recognition receptors (PRRs) expressed by the cells (150). Most recently, Kanmani and colleagues demonstrated that exopolysaccharides from *L. delbrueckii* regulates porcine innate immune response initiated by TLR3 activation in intestinal epithelia (151).

In conclusion, recent studies have indicated a relationship between intestinal microbiome composition and reduced efficacy of RV vaccine in developing countries (152, 153). Moreover, Ramakrishnan and colleagues hypothesized that other contributing factors may be involved in the reduced efficacy of RV vaccines seen in developing nations (154); hence further research is needed to demonstrate the role of gut microbiome in RV pathogenesis and transmission.

## Rotavirus Interactions With Innate Immune Receptors and Interferon Signaling

**Figure 2:**
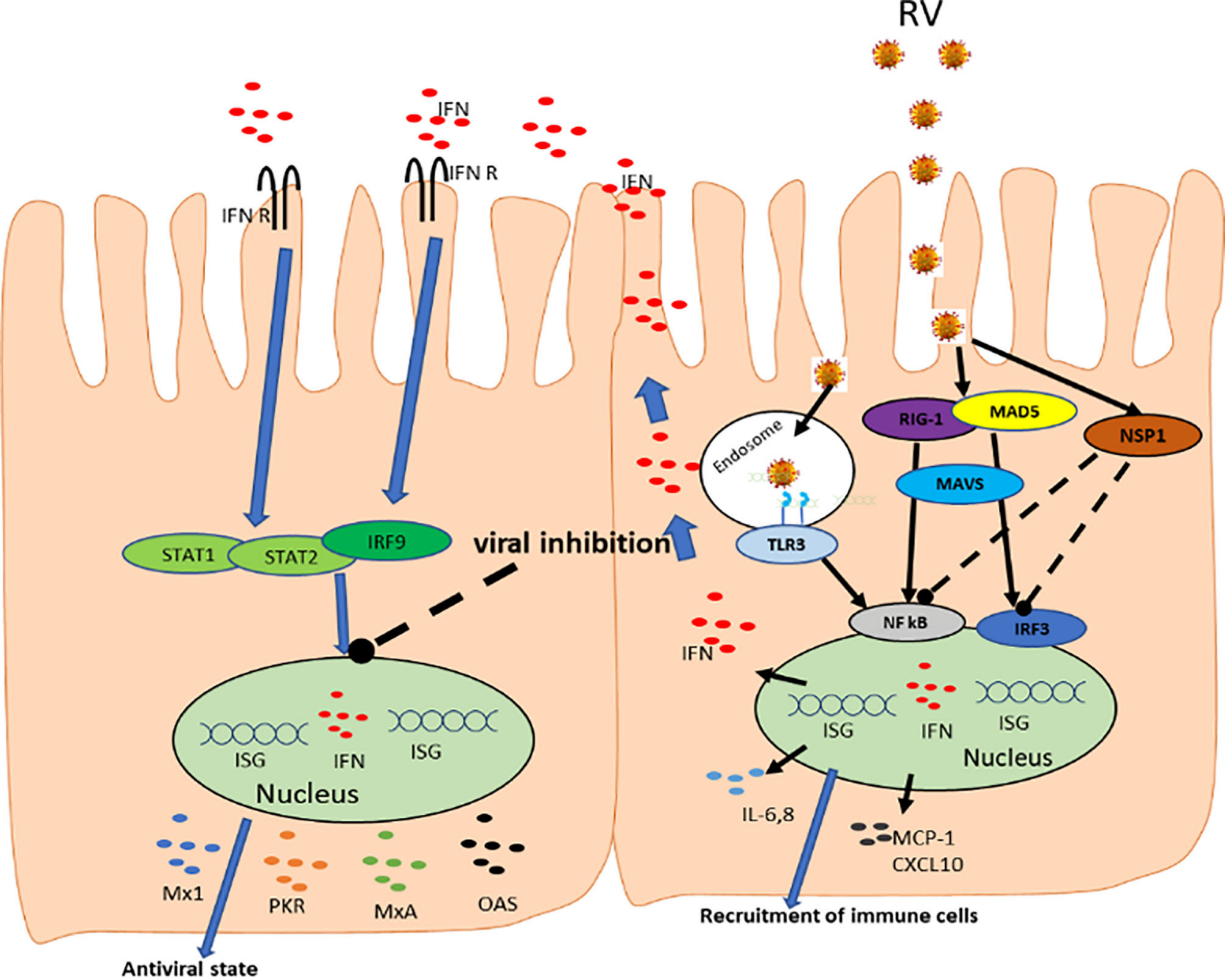
Rotavirus interactions with host innate immune system. RV enters cells, where it is recognized by RIG-I and MDA-5 receptors triggering the transcription factors IRF3 and NF-kB through signaling facilitated by MAVS. The activated IRF3 and NF-kB moves to the nucleus and upregulate the expression of type I and III IFNs which stimulates the synthesis of IFN stimulatory genes. Activation of NF-κB pathway by PRRs results in the production of proinflammatory cytokines and chemokines such as IL-6, IL-8, MCP-1, CXCL10. The IRF3 and NF-kB are degraded through interaction with viral NSP1. IFNs are then released and binds their receptors leading to activation of STAT-1, STAT-2, and IRF9 which further promote IFN production and creating ‘*antiviral state’*.

Viral infections are known to activate the host antiviral innate immune response, which is dependent on the recognition of virus by host PRRs, such as TLRs, RIG1-like receptors and melanoma differentiation-associated gene-5 (MDA-5) (**Figure 2**). Viruses on the other hand depend on host cells for their survival; hence, they have evolved various mechanisms to escape and/or weaken the host immunity, thereby manipulating host cell proteins for successful replication and transmission (9, 155)

**Figure 3:**
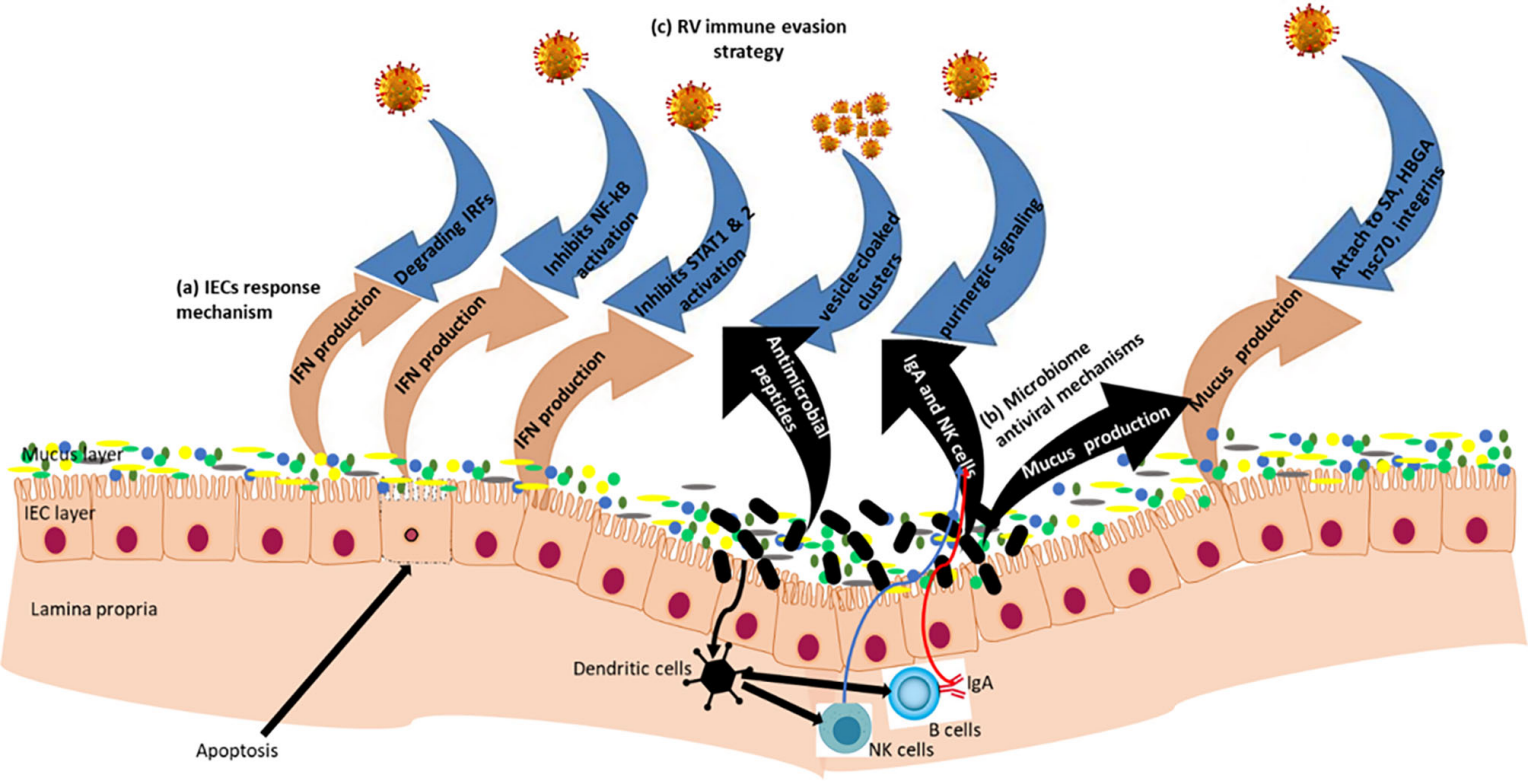
Rotavirus-IECs-gut microbiome Interactions: Gut epithelial surface covered by mucus layer containing glycoproteins (mucins) which provide a physical barrier between viruses and IECs. Mucin production is influenced by the gut microbiome composition of which some of the microbiota have antiviral properties. (a) In the IECs, RVs are recognized by the pattern recognition receptors (PRRs) and initiate mechanisms that induce IFN responses. (b) Beneficial microbiota inhibit viral attachment to host epithelial cells; they produce antimicrobial compounds which have antiviral effects; and also modulate innate and adaptive immune systems. (c) RV immune evasion strategy *via* blocking IFN responses through degradation of IFN Regulatory Factors (IRFs) and inhibition of nuclear accumulation of NF−κB, STAT1 and STAT2. RVs form pools of virions cloaked in extracellular vesicles to enhance multiplicity of infection, and RVs also utilize intercellular calcium waves and purinergic signaling to amplify intestinal pathophysiology.

Rotavirus infection rapidly triggers an innate immune response in the gut mucosa, during which type I and type III IFNs and other cytokines are produced limiting viral replication (**Figures 2**, **3**). Interferons are central to controlling RV infection and modulating the antiviral immune response. Virus-infected and noninfected (bystander) IECs, together with intestinal hematopoietic cells produce both type I and type III IFNs (156–159). These type I and III IFNs are the major part of the host defense mechanism against viruses, whereby lack of their receptors enhances susceptibility to virus infection (160, 161). Type III IFNs (IFN-λ) represent key innate immune barriers against viral infections with diverse immune and biological roles that may overlap and be distinct from those of type I IFNs (162). Type I IFNs in humans, are encoded by 13 genes for IFNα and one each for IFNβ, IFNϵ, IFNκ, and IFNω (163); while four genes encode for the type III IFNs: IFN-λ1 (IL-29), IFN-λ2 (IL-28a), IFN-λ3 (IL-28b) and IFN-λ4 (162, 164).

Upon antiviral response initiation, IFNα, IFNβ, IFN-λ are secreted by virus-infected IECs leading to the transcription of IFN-stimulated genes (ISGs). While type I and III IFNs and their receptors are not closely related, their signaling pathways are nearly identical. The antiviral protective role of either type I and type III IFNs depends on their receptor expression levels that significantly vary among different tissues defining their roles *in vivo* (165). Studies have shown that majority of cells can sense and respond to type I IFNs, whereas epithelial cells uniquely respond to type III IFNs (156, 166, 167). Although there is little published data on the specific cells capable of secreting IFN-λ, few studies have demonstrated that some cells may produce both IFN-λ and type I IFNs (164, 168, 169). The presence of IFN-λ and their receptors within IEC population supports a hypothesis that they could play a protective role in RV infection. Zou and colleagues in their review of the role of type III IFNs in viral infection and antiviral immunity concluded that IFN-λs provide antiviral protection at epithelial surfaces during the initial stages of viral infection. Moreover, these cytokines shift the Th1 and Th2 cells balance towards Th1 phenotype (162). Thus, the IFN-λ as a component of innate immunity might be involved in the adaptive immune response regulation.

Lin and colleagues (158) described distinct roles of type I and type III IFNs in gut immune response to RV infections in a mouse model, where they demonstrated that both IFNs are not efficient in inhibiting replication of murine RV. However, they showed that host adaptive immunity is involved in viral clearance. Similarly, the same authors demonstrated that only the presence of both type I and type III IFNs controls homologous murine RV replication and disease outcome in neonatal mice. Thus, their findings provided evidence that both type I and type III IFNs are essential for effective antiviral protection against the heterologous RRV infection in mice. Moreover, both IFNs play a key role in innate antiviral protection of the gut and they work together in preventing RRV replication outside the intestine. Similar to several other viral infections, RV host-range restriction is determined by the different efficiency of homologous versus heterologous RVs in interfering with the host IFN response. Pott and co-authors also demonstrated that RV infection of non-purified IECs prepared from mouse feces induces IFN-β and IFN-λ mRNA expression (170). In their study they observed that mice lacking the receptor for IFN-λ, and sometimes deficient of type I IFNs receptors, show increased levels of viral replication compared to their wild-type counterpart, further highlighting the key role of the IFN system in regulating viral replication (**Figure 1**). Sen et al. (159) demonstrated that type I IFN and IFN-stimulated genes can be transcribed by villous IECs, ultimately showing proof that RV actively counteracts both type I IFN synthesis and their effects in IECs.

Ding et al. using CRISPR/Cas9 approach demonstrated that loss of STAG2, a part of cohesin complex, confers resistance to RV replication in cell culture and human intestinal enteroids (82). Lack of STAG2 leads to gDNA damage and strong IFN expression resulting in JAK-STAT activation and ISG expression which protects against RV infections. Besides, Zang et al. showed that NOD-like receptor C4 (NLRC4) and TLR5 are important in anti-rotavirus host immune responses (171). TLR5 activation induced by Flagellin on DCs stimulated IL-22 production resulting in generation of protective gene expression system in IECs. Flagellin also stimulate the production of IL-18 through induction of NOD-like receptor C4 (NLRC4) thereby causing immediate removal of RV-infected cells (171). Recently, Zhu and colleagues demonstrated the role of NOD-like receptor P9b (Nlrp9b) in anti-rotavirus host immune response, whereby Nlrp9b restricts RV infection in IECs. They showed that depletion of NLRP9b in the intestine *in vivo* led to enhanced susceptibility of mice to RV replication (172).

### Rotavirus Mechanisms of Immune Evasion

As mentioned earlier in this review, RVs employ numerous strategies to evade host immune system and gut microbiota to ensure successful replication and transmission (**Figure 3**). Some of the mechanisms that have been identified and is discussed below include: (i) degradation of IFN-regulatory factors (15); (ii) inhibition of nuclear accumulation of STAT1, STAT2 (16) and nuclear factor κB (17, 18); (iii) formation of vesicle-cloaked virus clusters (19, 20); and (iv) induction of intracellular calcium waves through ADP signaling (21). Therefore, understanding mechanisms by which RVs evade host defense is crucial in the development of RV genotype specific or universal attenuated vaccines, or anti-rotaviral drugs targeting RV proteins acting as immune system antagonists.

#### Degradation of IFN-Regulatory Factors

As discussed above, interferons play an important role in anti-RV response. Rotaviruses, like other viruses, have coevolved with their hosts, acquiring different mechanisms to improve their survival and spread, including interrupting IFN-mediated responses in virus-infected or noninfected bordering IECs (**Figure 3**) (159, 173, 174). Broome and co-authors showed in mouse models that even less than 10 viral particles of a homologous RV leads to severe infection in animals having intact IFN response system (175). Therefore, besides inhibition of type I IFN secretion, RVs also suppress the late events of antiviral signals activated by dsRNA (155, 176, 177). Since IFNs plays a critical role in the development of an adaptive immunity, reducing or removing RV IFN antagonist activity might increase the post vaccination IFN response leading to higher level of adaptive immune response. Moreover, as shown in the previous section, HBGAs and other cell surface glycans also alter expression of some of the innate immune factors and therefore regulate RV infection (178).

As a major component of the immune system in the gut, IECs recognize pathogens by PRRs including TLRs that signal through IFN-Regulatory Factors (IRFs). These IRFs act as a bridge to the innate and adaptive immunity (15). Among the IRFs, IRF3 and IRF7 are the main modulators of type I IFN gene expression induced during RV infection. Following RV infection, phosphorylation of IRF3 serine residues occur in the cytoplasm to allow nuclear translocation (179). Likewise, IRF7 in the cytosol undergoes serine phosphorylation of its C-terminal region, permitting its dimerization and translocation to the nucleus (180). IRF3 activates IFN-β but not IFN-α (except IFN-α_4_), while IFR7 efficiently activates both the IFN-α and IFN-β genes (15, 181). Additionally, Honda and Taniguchi confirmed a key role of IRF5 in the production of proinflammatory cytokines *via* TLRs pathway (15). These proinflammatory cytokines are involved in earlier events of antiviral defense protection against RV (182).

Rotavirus uses NSP1 protein to interfere with host IFN response to successfully replicate and avoid elimination (**Figure 2**). Barro and Patton demonstrated that RVs through its NSP1 interferes with IFN signaling through degradation of both IRF3 and IRF7 (183). IRF7 degradation permits RV to move across the intestinal barrier, facilitating the virus to replicate in trafficking cells that express IRF7, such as dendritic cells and macrophages. Additionally, together with IRF3 and IRF7, NSP1 also triggers the degradation of IRF5 thereby regulating apoptosis during viral infection. NSP1, therefore, is a broad-spectrum antagonist of IRF function. Remarkably, NSP1s from animal RVs degrades IRF3, IRF5 and IRF7; however, NSP1s of human RVs primarily degrade IRF5 and IRF7 and therefore, inhibit the IFN response less efficiently compared to animal RVs (184). Degradation of IRF by NSP1 may lead to downregulation of the activity of genes responsible to produce proinflammatory cytokines that stimulate the sequence of events resulting in apoptosis. Hence, NSP1 driven inhibition of the apoptosis of host cells allows RV to persist longer in infected cells. Thus, these effects of NSP1 on innate immune response and virus spread led to the conclusion that NSP1 is a key factor determining RV virulence (183, 185, 186). Moreover, Arnold and Patton observed that while IRFs degradation is well-known characteristic among wild-type RVs, however, one RV strain did not exhibit this activity (184). Studies have also revealed that NSP1 is also involved in degradation of other host immunity components in strain-specific manner (187–189). Additionally, NSP1 protein of some animal RVs can inhibit the transcriptional activity of IRF3 without causing its degradation (190) or block the signaling of RIG-1 (191) and MAVS (192) (**Figure 2**). These observations implies that NSP1 has employs different strategies to inhibit the activation of IFNs and ISGs in the host epithelial cells (14, 193, 194) and also for blocking the IFN signaling in neighboring noninfected IECs (165). More recently, Iaconis and coworkers showed that NSP1 from many human and animals RV strains is a potent inhibitors of IRF1 than either IRF3 or IRF7 thereby antagonizes type I and III IFN production (195). Ding et al. demonstrated that RV uses its VP3 gene to degrade MAVS in a host restricted manner leading to inhibition of type III IFN expression in IECs (196). Additionally, studies have demonstrated important role of VP3 during RV replication (197–199). The 2`-5`-oligoadenylate synthetase (OAS)/RNase L pathway is activated by innate immunity during RV infection where it blocks RV replication in a strain-specific manner (198). However, RV uses two strategies to evade this effect, including i) inhibition of RNase L activity through initial interaction of viral particles and the cells early during virus entry; ii) the phosphodiesterase activity of VP3 degrades the cellular OAS, potent activators of RNase L, preventing its activation (197). *Therefore, both the inhibition of production of proinflammatory cytokines and blocking of apoptosis are key strategies of RV immune evasion.*

#### Inhibition Accumulation of STAT1, STAT2 and NF-κB in the Nuclear

NF-κB is required for the secretion of IFNs and chemokines which function as antivirals (17, 18). NF-κB also plays a role in inhibition of apoptosis and mediates proliferation of epithelial cells, which benefits the virus since apoptosis - a key host defense strategy for the elimination of virus-infected epithelial cells (186, 200). Therefore, activation of NF-κB to prevent the cells from undergoing apoptosis is a key viral immune evasion strategy employed by RVs to ensure survival. However, in the initial stages of infection, to prevent viral clearance, RVs temporarily inhibit NF-κB activation to delay initiation of the innate immune responses until they establish the infection (16, 155). Graff et al. (186) reported stability of phosphorylated inhibitors of κBα in RV-infected cells due to induction of proteasome-dependent degradation of β-transducin repeat containing protein (β-TrCP) further confirming that RV NSP1 inhibits NF-κB activation as immune evasion strategy. Previous studies have demonstrated that degradation of β-TrCP by NSP1 was mediated by the host cullin-3 E3 ligase complex, an essential multisubunit ubiquitination complexes (201, 202). Moreover, they showed that NSP1 may use this complex to prevent other cellular activity, hence a need for more studies to unravel how NSP1 subverts the host IFN response.

The mechanisms utilized by RV to inhibit accumulation of STAT1 and STAT2 and their functions leading to inhibition of all types of IFN responses in the IECs are still not known (16). This immune evasion strategy was reported by Holloway and co-workers for rhesus RV (RRV) and human RV Wa strains, even though the underlying mechanism of the observed inhibition was not confirmed. Since it is well established that nuclear NF-κB accumulation is inhibited by NSP1(**Figure 2**), and that NF-κB inhibits apoptosis, it is clear that inhibition of NF-κB by NSP1 should lead to enhanced apoptosis of infected IECs. Finally, Jiang et al. further demonstrated that binding of NSP2 to 3′-end of RV mRNA is another immune evasive mechanism utilized by RVs (203). Altogether, these findings confirm that IFN-signaling cascade triggered by RV infections is a complex process. Therefore, more studies are needed to investigate whether RV NSPs can interact with other signaling molecules to evade IFN-responses. Further work will define the activity of NSP1 in order to clarify the role of RVs IFN antagonists in supporting viral persistence and transmission.

#### Rotavirus Exploit Intercellular Calcium Waves and Purinergic Signaling to Amplify Intestinal Pathophysiology

RV infection of enterocytes and enteroendocrine cells in the small intestine results in the disruption of host cell calcium signaling. RV induces changes to the cell homeostasis resulting in the secretion of paracrine signaling molecules such as enterotoxin NSP4, prostaglandin E2, and nitric oxide (21). Studies have shown that during RV infection, infected cells may crosstalk with neighboring uninfected cells *via* a purinergic signaling pathway (21, 204), resulting in the induction of intercellular calcium (Ca^2+^) waves (ICWs) that disseminate among infected and adjacent bystander cells. RV-infected cells secret serotonin in response to purinergic signaling leading to diarrhea, and therefore, diarrhea severity could be reduced by inhibition of purinergic receptors (P2Y1). Chang-Graham et al. (21) demonstrated the role of NSP4 in RV infection, where they showed that NSP4 stimulates Ca2^+^-activated chloride channels by binding neighboring noninfected enterocytes resulting in secretory diarrhea. However, in the same experiment they demonstrated that ICW spread to adjacent uninfected cells is achieved by ADP acting on P2Y1 receptors, and does not require NSP4. Thus, they observed that purinergic process was essential for RV replication rate and stimulation of secretion of cellular factors critical in the development of pathology associate with RV infection. Therefore, blocking purinergic signaling lowers secretion of fluids in IECs and decreases RV-mediated serotonin production. Serotonin is a major neurotransmitter in intestinal physiology, causing severe water loss within IECs leading to diarrhea (3). In their study, Hagbom and colleagues observed decreased diarrhea severity and short recovery time in suckling mice when P2Y1 receptors were inhibited. Collectively, expanding infectious disease research to include both infected cells and adjacent noninfected cells would expand our understanding of disease progression and accordingly, lead to development of effective therapeutics and preventative strategies including vaccines.

#### RV Uses Vesicle-Cloaked Virus Clusters Strategy to Enhance Multiplicity of Infection, Disease Severity and Evade Immunity

Most viruses have developed multiple strategies to enhance chances of each viral unit to cause effective infections. One known strategy involves spreading in vesicle-cloaked clusters to increase the multiplicity of infection at the cellular level (205). Several mechanisms that viruses use to achieve this includes virion load guided by specific extracellular components, hiding within lipid vesicles, encapsulated in protein matrices, or adhering to microbiota and host surface cells (19). There are numerous benefits in viruses spreading in clusters; (i) “mass effect” where the likelihood of successful infection increases in cells receiving multiple genome copies, (ii) “heterotypic cooperation” whereby the advantage of collective infection resides precisely in bringing together different genetic variants; and (iii) evasion of circulating antibodies (19, 205, 206). However, spreading in clusters may reduce dispersal and encourage evolution of variants that benefit from others without reciprocating.

Non-enveloped enteric viruses, including RVs, protect themself from host immune degradation by leaving the infected cells before lysis in pools of virions covered by extracellular matrix, hence increasing their infectivity (20, 206, 207). Studies have demonstrated that pooled viruses have larger contribution to the infectivity of stool than free virus particles, suggesting that viruses in groups are extremely infective units (20). The findings by Santiana and co-authors demonstrated that vesicle-cloaked viruses and not free viruses are more effective in fecal-oral transmission and dramatically increase multiplicity of infection upon contact with susceptible cells. Thus, spreading in clusters or the mixture of free virions and group dispersal is beneficial to the RVs; however, limited data are available addressing the mechanisms involved. Thus, more research is needed to verify whether viral spreading in clusters has a genetic basis and if so, there is a need to distinguish mutants that creates that trait. Overall, these findings emphasize the need for developing antiviral therapies capable of counteracting viral clustering mechanisms for effective control of RV infections and spread.

## Conclusions and Future Perspectives

Knowledge of the interactions between a cellular host and viral pathogen is crucial for resolving membrane cell trafficking and for understanding epithelial pathology, with clear significance for designing antiviral strategies. Furthermore, understanding mechanisms of RV entry into the cell is essential for optimal design of live virus vaccines since initial RV-IEC interactions affects viral pathogenesis as well as the host immunity.

Since IFNs are essential for development of adaptive immunity, reduction or elimination of viral IFN antagonist activity might stimulate production of more IFNs upon vaccination, leading to a more effective adaptive immunity. Optimization of vaccine informed by both host and RV genetics should be prioritized, since the efficacy of current vaccines in the low resource countries (high RV prevalence) due to RV infections is suboptimal compared to that in developed countries.

Many studies have shown that all globally dominant RV genotypes carry highly conserved glycan binding sites. Thus, the conserved site could be ideal target region for developing effective antiviral therapeutics and formulation of vaccines.

An improved understanding of how host glycan profiles affect host immunophenotypes and responses to RV would allow identification of at-risk populations and novel therapeutic targets. Furthermore, the latest knowledge of HBGA-controlled RV host ranges in different populations and susceptibility to RV infection based on age, have considerably expanded our knowledge on RV epidemiology.

An in depth understanding of the interactions between the gut microbiota and innate immunity is invaluable for designing probiotic formulations capable of reducing mortality and/or severity of RVs disease. However, more studies are required to determine to the role of HBGA-like-coated bacteria and free HBGA-like substances in RV infection.

Finally, a better understanding of mechanisms by which RVs evade host defense is crucial in the development of RV genotypes specific attenuated vaccines, and antiviral therapies.

## Author Contributions

JA and AV conceived the idea and drafted the manuscript. JC, SR, AM, and YG collected some of the literature and contributed in some sections. JA, LS, and AV edited and revised the manuscript. All authors contributed to the article and approved the submitted version.

## Funding

This work was supported in part by the International Development Research Centre, Canada, (grant #109053 to AV; https://www.idrc.ca/).

## Conflict of Interest

The authors declare that the research was conducted in the absence of any commercial or financial relationships that could be construed as a potential conflict of interest.

## Publisher’s Note

All claims expressed in this article are solely those of the authors and do not necessarily represent those of their affiliated organizations, or those of the publisher, the editors and the reviewers. Any product that may be evaluated in this article, or claim that may be made by its manufacturer, is not guaranteed or endorsed by the publisher.
